# Cohesin promotes genomic stability by suppressing unequal sister chromatid exchange

**DOI:** 10.64898/2026.07.31.742155

**Published:** 2026-08-10

**Authors:** Vincent Guacci, Nicola Minchel, Tik Sung, Sergey Venev, Job Dekker, Douglas Koshland

## Abstract

The protein complex cohesin plays critical roles in genomic stability by tethering together sister chromatids at their pericentric regions and along their arms from S phase until anaphase. Cohesin-mediated pericentric cohesion prevents aneuploidy by ensuring bipolar attachment of sister kinetochores. Arm cohesion prevents loss of heterozygosity by biasing DNA repair via recombination between sister chromatids rather than between homologs. Here, we investigate in yeast whether cohesin also enhances genomic stability by suppressing unequal sister chromatid exchange (USCE) between repetitive sequences. In wild-type cells, the USCE rate between repeats 4kb apart (proximal) was 15X higher than repeats 68kb apart (distal). The USCE between distal repeats but not proximal repeats increased 4 to 7-fold in mutants with altered cohesin subunits or auxiliary factors. The level of increased distal USCE corresponded with reduced arm cohesion, reduced density of cohesion arm sites, and higher sister loci mobility. Our results suggest that high density of arm cohesion sites confines repair of DNA damage to local sequences. When the density of cohesion sites decreases, sister chromatid sequences are less confined, thereby enhancing distal repeat interactions and USCE. Another set of mutations disrupted both DNA replication and cohesin loading at the replication fork during S phase. Remarkably, distal USCE in these mutants increased approximately 100-fold and was 6-fold more likely than proximal USCE. This preferential hyperdistal USCE can be explained by an aberrant sister-chromatid structure that is normally prevented by proper coupling of cohesin function and replication.

## Full Text Availability

The license terms selected by the author(s) for this preprint version do not permit archiving in PMC. The full text is available from the preprint server.

