# Supplementary material for: Cohesin promotes genomic stability by suppressing unequal sister chromatid exchange
Source: bioRxiv. 2026 Sep 1:2026.07.31.742155. Preprint. [Version 3] doi: 10.64898/2026.07.31.742155 (PMC13484563; doi:10.64898/2026.07.31.742155)
Supplement: Supplementary file 1 [file NIHPP2026.07.31.742155V3-supplement-1.pdf]

bioRxiv preprint doi: https://doi.org/10.64898/2026.07.31.742155; this version posted September 1, 2026. The copyright holder has placed this preprint (which was not certified by peer review) in the Public Domain. It is no longer restricted by copyright. Anyone can legally share, reuse, remix, or adapt this material for any purpose without crediting the original authors.

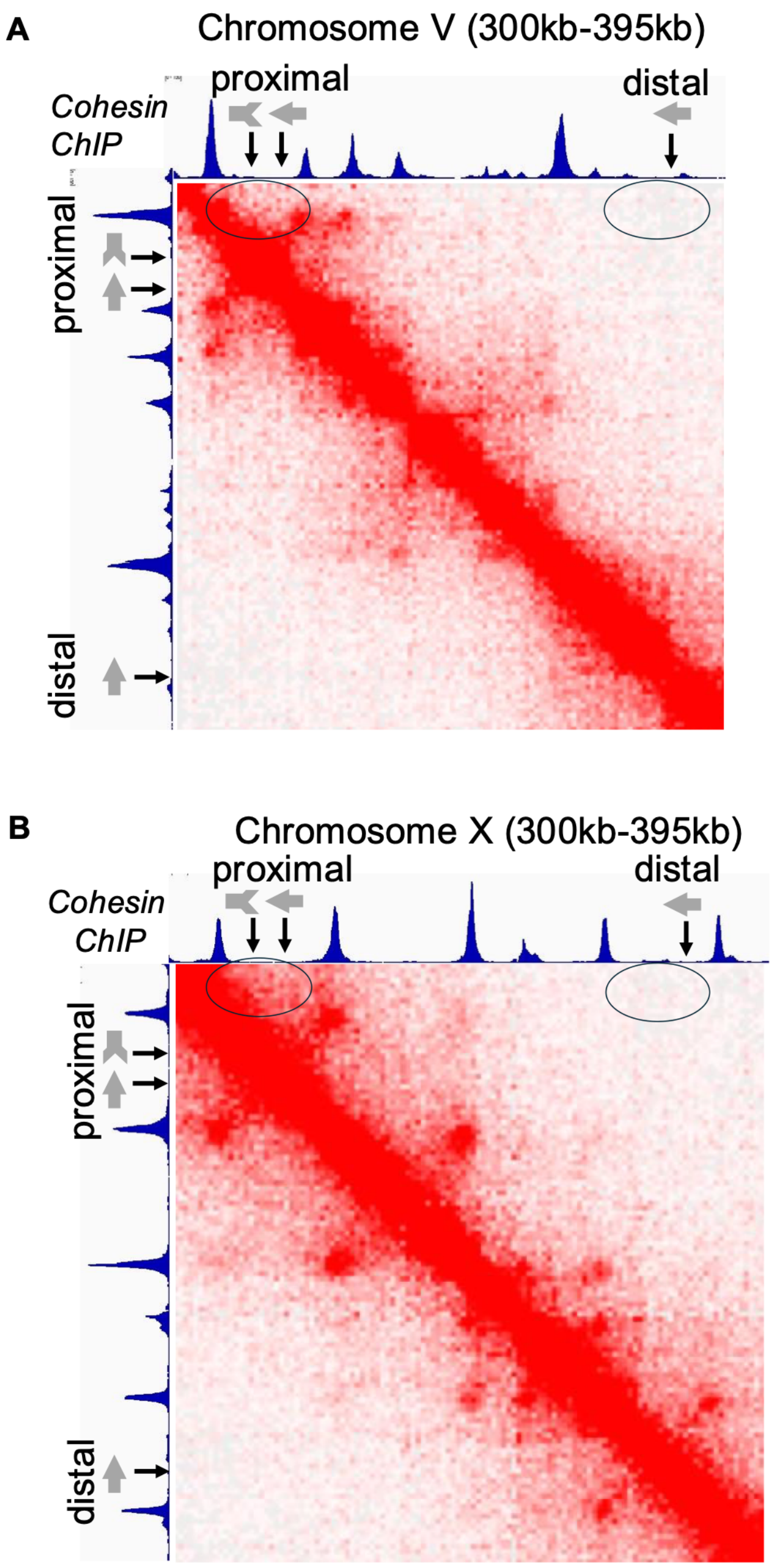

Fig. S1. MicroC maps of chromosomes V and X showing where hyg fragments were inserted for uSCE assays and interactions in proximal regions or distal regions. (A-B) MicroC of WT yeast arrested in mid-M phase. A) Chromosome V right arm map from 300kb to 395kb with cohesin peaks defined by Mcd1p ChIP (blue peaks) Shown are the sites where the 3'hyg fragment (grey arrow tail) and the the 5'hyg fragments (grey arrowhead) at proximal (4kb) or distal (68kb) site, respectively. MicroC map of this region (red). Red dots off the main red axis show where adjacent DNA sites containing cohesin peaks interact thereby looping out intervening DNA. The black oval on the upper left shows interactions of DNA within the proximal region. The black oval on the upper right shows an absence of DNA interactions between the proximal and distal regions. B) Chromosome X left arm from 300kb to 395kb with hyg fragment insertion sites, Mcd1p ChIP and MicroC as described in (A). (C-D) MicroC of *wpl1Δ* yeast arrested in mid-M phase. C) Chromosome V as displayed as described in (A). D) Chromosome X as displayed as described in (B).

bioRxiv preprint doi: https://doi.org/10.64898/2026.07.31.742155; this version posted September 1, 2026. The copyright holder has placed this preprint (which was not certified by peer review) in the Public Domain. It is no longer restricted by copyright. Anyone can legally share, reuse, remix, or adapt this material for any purpose without crediting the original authors.

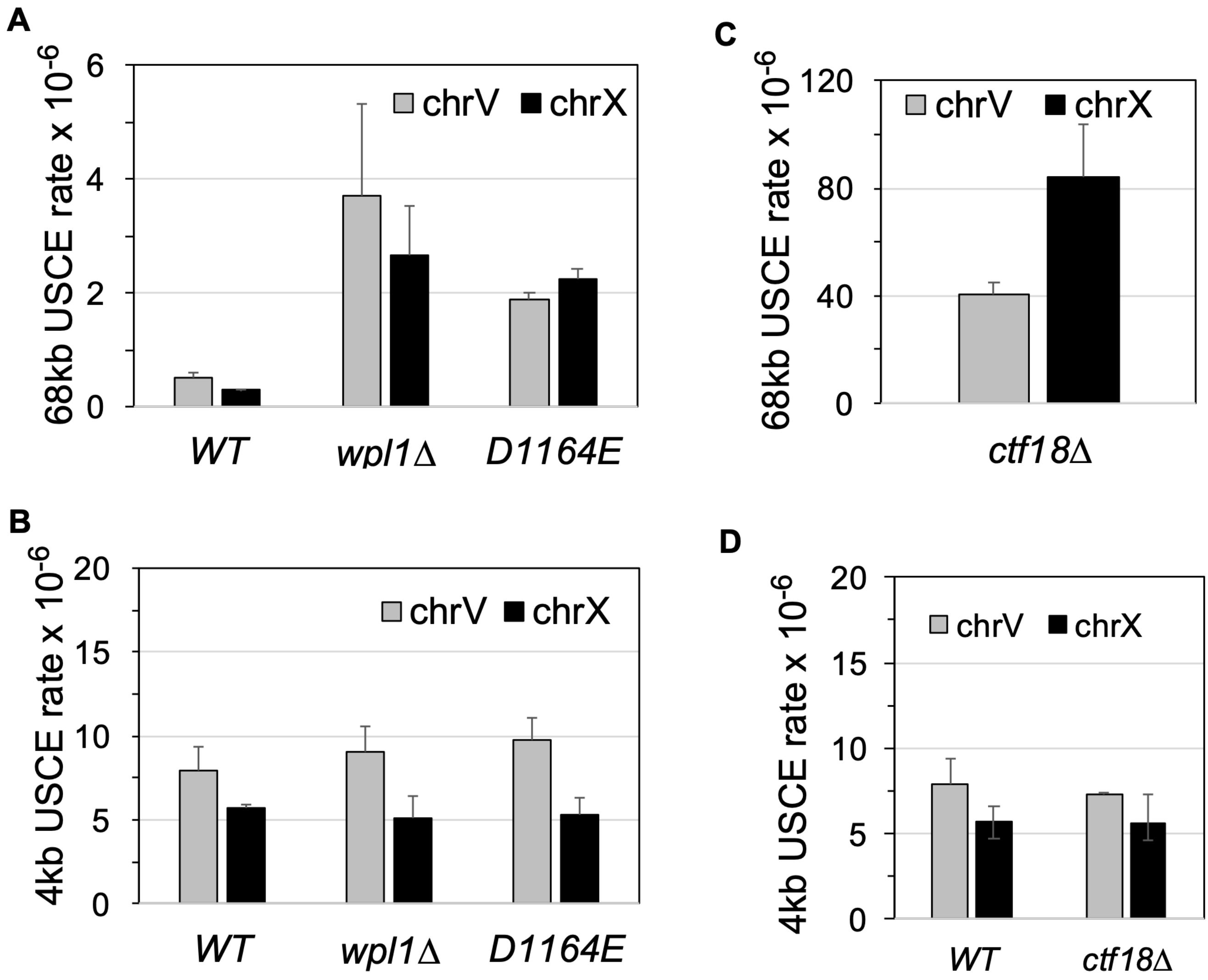

Fig. S2. Mutants have similar USCE rates on chromosomes V & X. USCE rates for chromosome V (grey bars) and for chromosome X (black bars) are shown **(**A & C) Distal USCE for chromosomes V & X. Chromosome X uSCE data was generated using strains VG4346-1A (wild-type; WT), VG4364-5C (*wpl1Δ*), VG4363-4D (*smc1-D1164E*; DE) and VG4362-2B (*ctf18Δ*) as described (Materials and methods). Data for chromosome V was taken from Figures 3B, 3C & 6A and replotted for comparative purposes. (B) Proximal USCE for chromosomes V & X. Chromosome X uSCE data was generated using strains using strains VG4460-13C (wild-type; WT), VG4466-13B (*wpl1Δ*), VG4463-4D (*smc1-D1164E*; DE) and VG442-2B (*ctf18Δ*) as described (Materials and methods). Data for chromosome V was taken from Figures 3C and 6B and replotted for comparative purposes. Data for chromosome X was from 2 independent experiments.

bioRxiv preprint doi: https://doi.org/10.64898/2026.07.31.742155; this version posted September 1, 2026. The copyright holder has placed this preprint (which was not certified by peer review) in the Public Domain. It is no longer restricted by copyright. Anyone can legally share, reuse, remix, or adapt this material for any purpose without crediting the original authors.

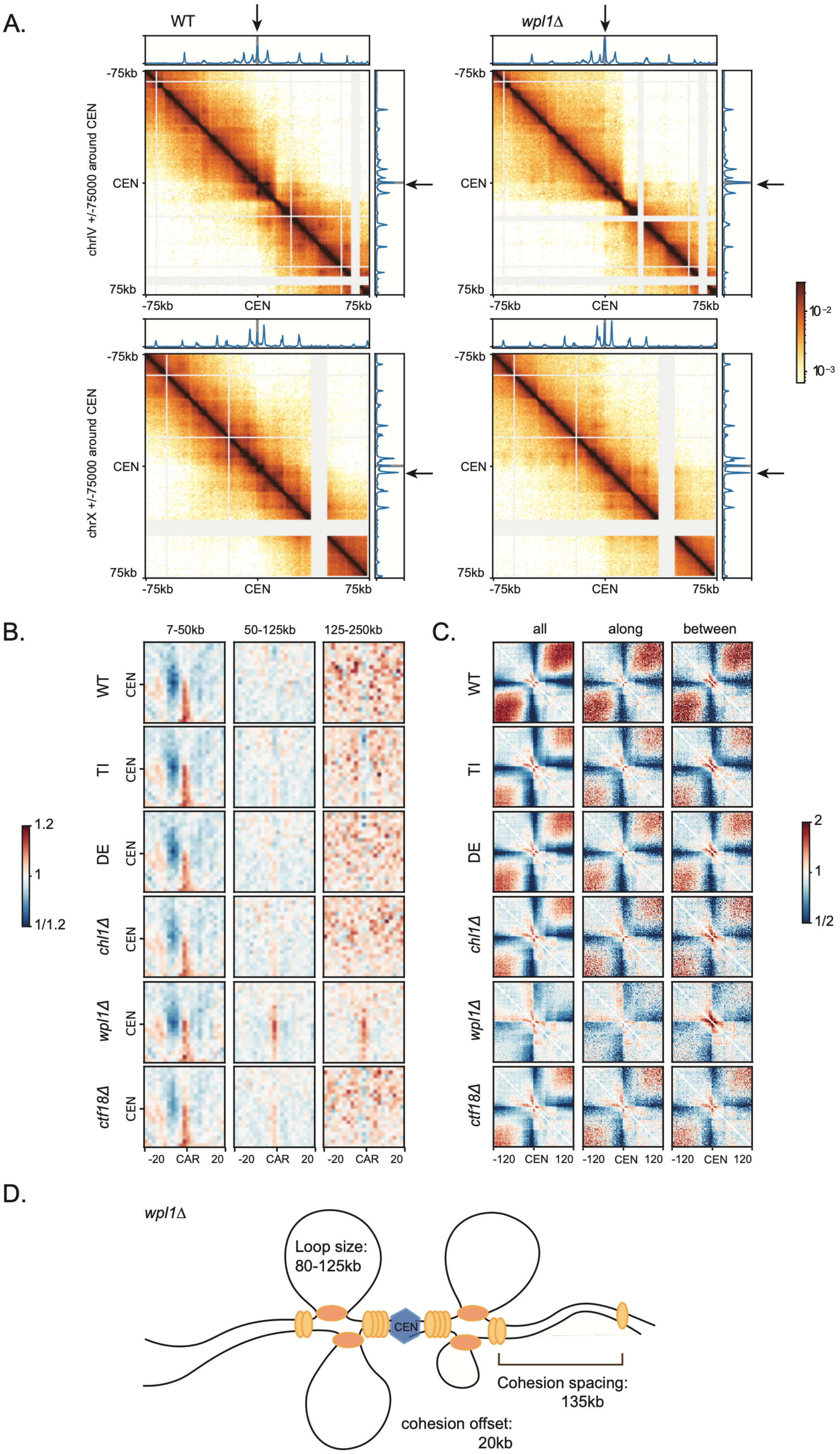

bioRxiv preprint doi: https://doi.org/10.64898/2026.07.31.742155; this version posted September 1, 2026. The copyright holder has placed this preprint (which was not certified by peer review) in the Public Domain. It is no longer restricted by copyright. Anyone can legally share, reuse, remix, or adapt this material for any purpose without crediting the original authors.

Fig. S3. Large loops in *wpl1Δ* arise from centromeric cohesin. (A) Representative Hi-C maps plotted at 1kb resolution with Rad21 chip-seq tracks from chromosomes 4 and 10 in wild-type and *wpl1Δ* cells. The arrows indicate centromeric cohesin loops visible in *wpl1Δ* compared to WT. (B) Pile-up interaction maps based on one cohesin chip signal at the centromere with all arm CARs; plotted by distance from the centromere at a 2kb resolution. (C) peri-centromeric pile-ups of all interactions, intra-sister [along] interactions, and inter-sister [between] interactions. Plotted at a 2kb resolution. (D) Schematic representing the effects on sister chromatids of cohesive and extruding cohesin in a *wpl1Δ* background.

Chip-seq of *wpl1Δ* showed increased binding of cohesin around centromeres. The centromeric and peri-centromeric CARs appeared to have increased interactions with arm CAR's showing visible spots on the heatmap (black arrows). When we piled up these interactions based on one centromeric CAR with all arm CAR sites we found that all the strains tested had interactions from the centromeric CAR to arm CAR sites up to 50 kb as expected. However, *wpl1Δ* had strong visible interactions up to 250 kb whereas no other strains we looked at had these interactions (Supp Figure NM B). We also noted an increase in trans cen-cen interactions in the *wpl1Δ* strain perhaps indicating strong cohesion at the centromere compared to the arm regions (supp Figure NM C). Overall, most cohesin being anchored at the centromeres, keeping centromeres cohesed, while being depleted along chromosome arms.

bioRxiv preprint doi: https://doi.org/10.64898/2026.07.31.742155; this version posted September 1, 2026. The copyright holder has placed this preprint (which was not certified by peer review) in the Public Domain. It is no longer restricted by copyright. Anyone can legally share, reuse, remix, or adapt this material for any purpose without crediting the original authors.

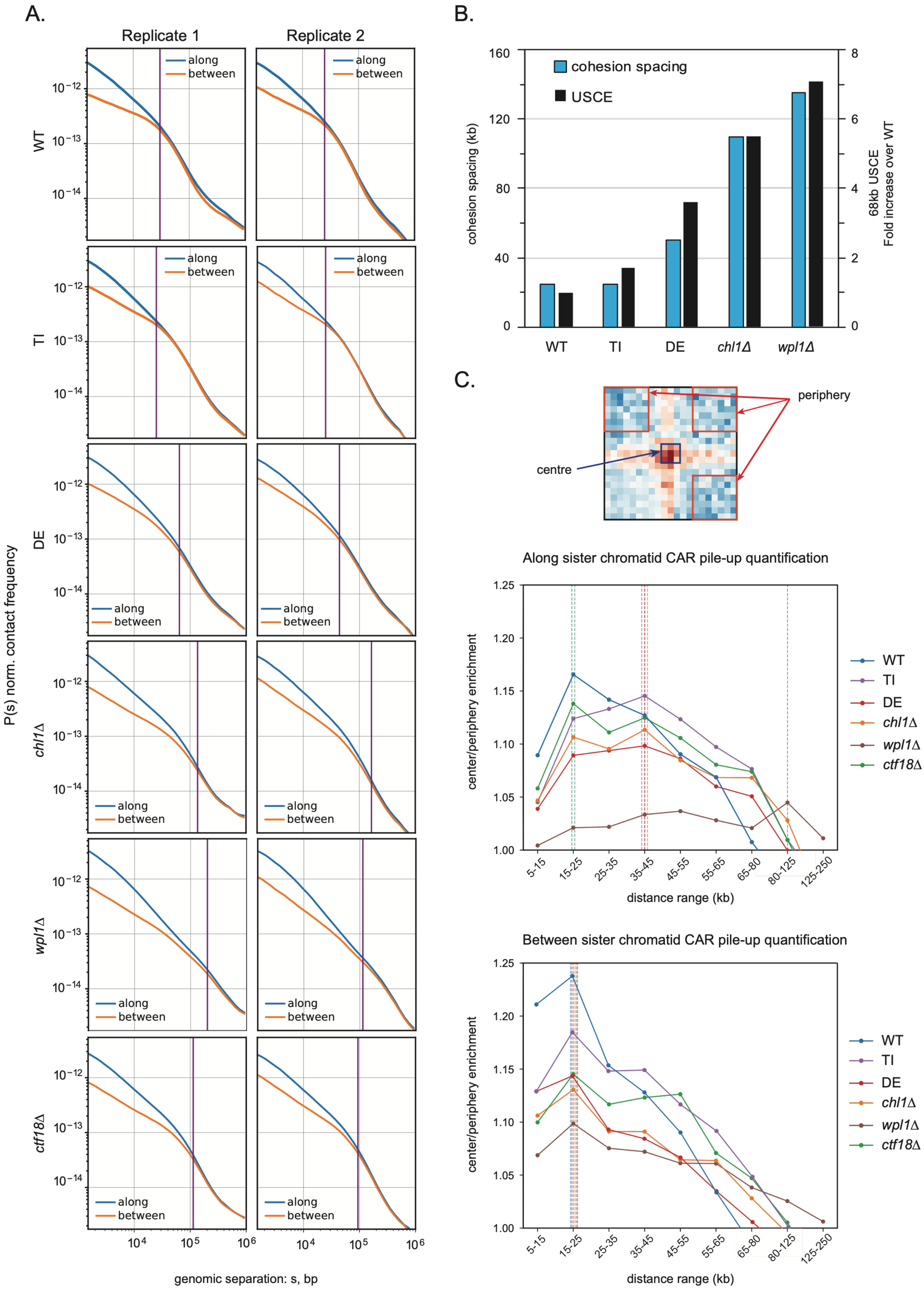

bioRxiv preprint doi: https://doi.org/10.64898/2026.07.31.742155; this version posted September 1, 2026. The copyright holder has placed this preprint (which was not certified by peer review) in the Public Domain. It is no longer restricted by copyright. Anyone can legally share, reuse, remix, or adapt this material for any purpose without crediting the original authors.

Fig. S4. Sister-C replicates and quantification. (A) Contact frequency *P* plotted as a function of genomic separation for interactions either along or between sister chromatids. Two replicates shown that were combined in Figure 4A. (B) Dual axis histogram showing the 68 kb USCE fold increase (secondary y-axis) against cohesion spacing (primary y-axis) (from Figure 4A) for WT, TI, DE, *chl1Δ* and *wpl1Δ*. (C) Quantification of Figure 4B and 4C pile-up interaction maps. Inter-CAR interaction enrichment was calculated by normalising the average of the centre 3x3 pixels with the average of the peripheral 7x7 pixels from the three quadrants shown. Enrichment values for each distance shown for each strain. Dotted lines represent the strongest enrichment peak for each strain.

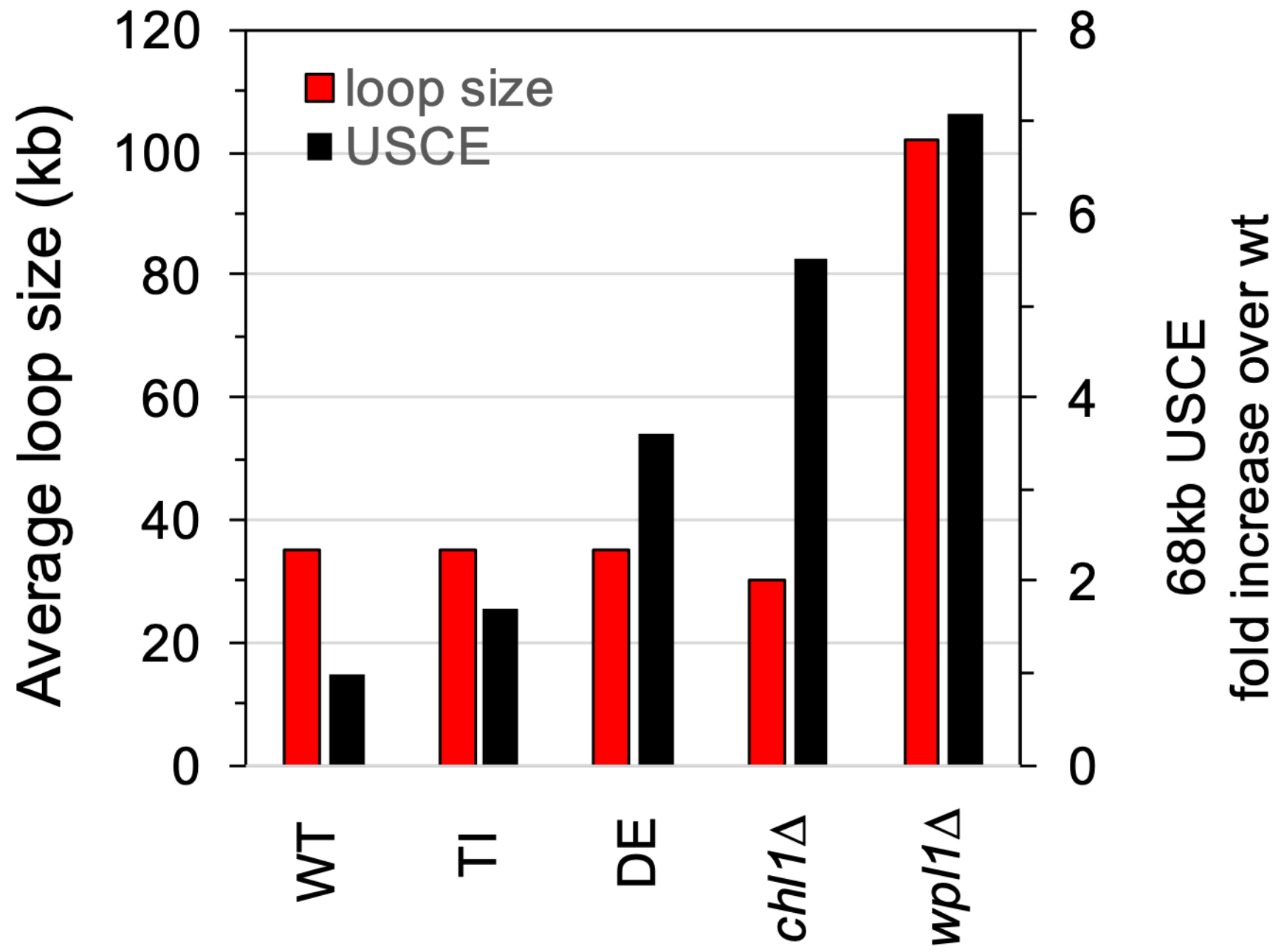

Fig. S5. Loop size does not correlate with increased distal USCE rates seen in cohesin mutants. Comparison of average loop size of haploid wildtype strain [WT] and mutant haploids [TI], [DE], [*chl1Δ*] and [*wpl1Δ*] taken from Fig. 4D plotted against the fold increase of distal USCE in each mutant taken from Fig. 3A calculated by dividing the rate of distal USCE in mutants by the rate for WT.

bioRxiv preprint doi: https://doi.org/10.64898/2026.07.31.742155; this version posted September 1, 2026. The copyright holder has placed this preprint (which was not certified by peer review) in the Public Domain. It is no longer restricted by copyright. Anyone can legally share, reuse, remix, or adapt this material for any purpose without crediting the original authors.

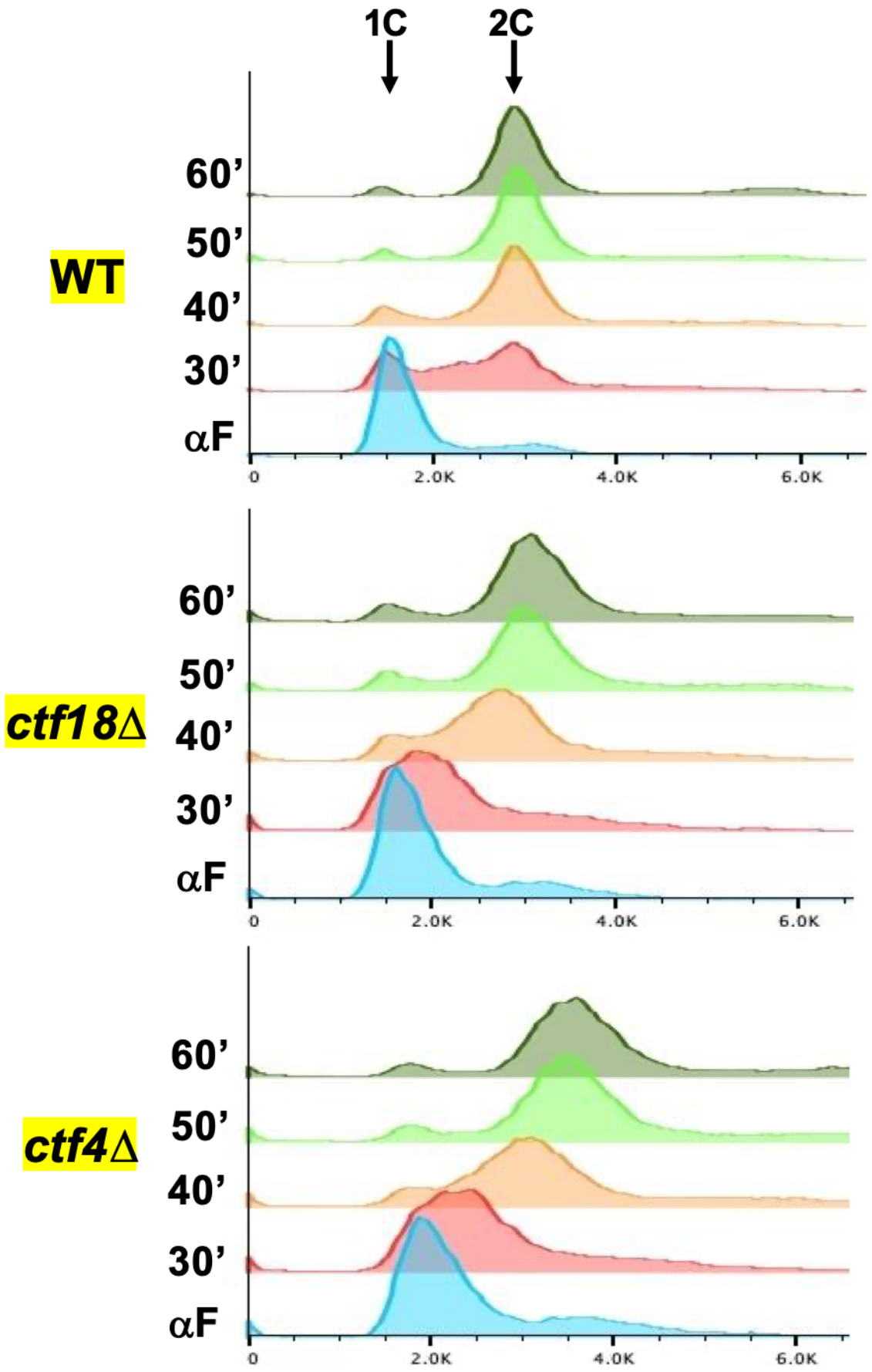

Fig. S6. *ctf4Δ* and *ctf18Δ* mutants exhibit a DNA replication delay. Haploid WT (VG3620-4C), *ctf4Δ* (SX347) and *ctf18Δ* (VG4402-3C) were arrested in G1 phase and synchronously released from G1 and arrested in mid-M phase as described in materials and methods, except arrest was for 3h instead of 2h. Cells were fixed and processed for FACS (Materials and methods).

bioRxiv preprint doi: https://doi.org/10.64898/2026.07.31.742155; this version posted September 1, 2026. The copyright holder has placed this preprint (which was not certified by peer review) in the Public Domain. It is no longer restricted by copyright. Anyone can legally share, reuse, remix, or adapt this material for any purpose without crediting the original authors.

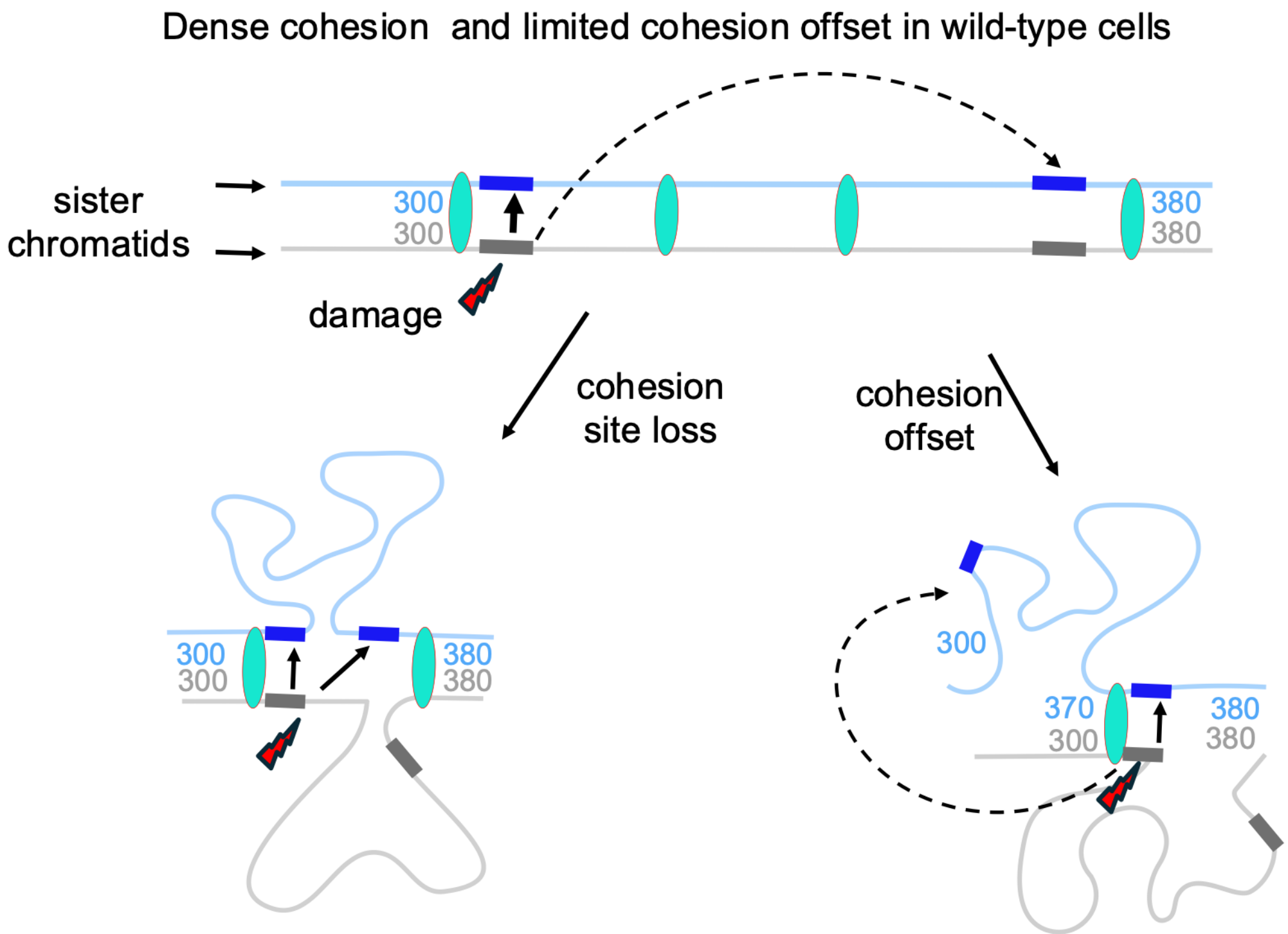

Fig. S7. Model for cohesin dependent suppression of distal USCE. A pair of sister chromatids (blue and grey lines) are held together by cohesin (green oval). Blocks represent a homologous repeat sequence present at 300 and 370 kb. Damage to the grey repeat at 300 kb is repaired primarily using the blue repeat at the 300 kb (solid arrow) because this repeat is held in close proximity to the damaged repeat by cohesin (top). Dense cohesion also limits loop extrusion, preventing cohesin from bringing together the damaged repeat with the 370 kb repeat (not shown). Loss of cohesion sites allows the repeats at 300 kb to diffuse away from each other (bottom left). This improves chances for damaged 300 kb repeat to reach the repeat at 370 kb either diffusion or cohesin-dependent loop extrusion (hashed green oval). Alternatively cohesion offset occurs between sequences 300 kb on the grey sister chromatid and 370 kb of the blue sister chromatid (bottom right). When the repeat at 300 kb is damaged it is preferentially repaired by the repeat at 370 kb due to its greater proximity.
